# Ulcération chronique sur la main d’un mécanicien: penser au carcinome épidermoïde

**DOI:** 10.11604/pamj.2017.28.141.13595

**Published:** 2017-10-14

**Authors:** Ilhame Naciri, Baderddine Hassam

**Affiliations:** 1 Service de Dermatologie et Vénérologie, Centre Hospitalier Universitaire IBN Sina, Faculté de Médecine et de Pharmacie, Université Mohammed V, Rabat, Maroc

**Keywords:** Carcinome épidermoïde, mécanicien, kératose actinique, Squamous cell carcinoma, mechanic, actinic keratosis

## Abstract

Cutaneous squamous cell carcinoma is a malignant tumor developed from the epidermis or the squamous mucosa. It may occur de novo or, most often, on precancerous lesions, including actinic keratoses. This tumor can sometimes be secondary to physical or chemical hazardous conditions encountered during the professional activity. We here report the case of a 40-year old mechanic presenting with ulceration of the dorsal side of his right wrist, evolving over 6 months. The lesion had first appeared as a small keratosic lesion that had transformed into an erosion and then into an ulceration increasing rapidly in size. The patient had no initial trauma and he had no particular previous history except the handling of chemicals (fuels, mineral oil, paint) without gloves for about the last 30 years. Physical examination showed a large sized ulcero-budding tumor (5 × 6 cm) on the dorsal side of his right wrist (A), associated with diffuse multiple actinic keratosis lesions on his two forearms and on the back of his hands (B). The patient also had two painless hard mobile adenopathies measuring 1.5 cm in diameter, on the ipsilateral epitrochlear and axillary regions. The remainder of the clinical examination was normal. Viral hepatitis serology test, treponemal test and retrovirus (HIV) test were negative. Histological examination of a biopsy sample confirmed the diagnosis of well differentiated and infiltrating squamous cell carcinoma. The biopsy of the axillary adenopathy objectified ganglionic metastasis with capsular break-in and extension to the periganglionar tissue. The remainder of the staging was without abnormalities. The patient underwent wide resection of the lesion with lymph node dissection followed by radiation therapy. Patient’s evolution was marked by early local recurrence (two months later) requiring reoperation. The postoperative course was uneventful.

## Image en médecine

Le carcinome épidermoïde cutané est une tumeur maligne développée à partir de l’épiderme ou des muqueuses malpighiennes. Il peut survenir de novo, ou le plus souvent sur des lésions précancéreuses, notamment les kératoses actiniques. Cette tumeur peut parfois être secondaire à des nuisances physiques ou chimiques rencontrées lors de l’exercice professionnel. Nous rapportant le cas d’un patient âgé de 40 ans, mécanicien, qui consultait pour une ulcération de la face dorsale du poignet droit, évoluant depuis 6 mois. La lésion avait débuté par une petite lésion kératosique, qui s’était transformée vers une érosion, puis une ulcération rapidement augmentée de taille. Le patient n’avait pas de notion de traumatisme initial, et il n’avait aucun antécédent particulier en dehors de la manipulation de produits chimiques (carburants, huile minérale, peinture), sans port de gants depuis environ 30 ans. L’examen physique trouvait une tumeur ulcéro-bourgeonnante de grande taille (5 × 6 cm) sur la face dorsale du poignet droit (A), associée à de multiples lésions de kératose actinique diffuses sur les deux avant-bras et le dos des mains (B). Le patient avait également deux adénopathies fermes mobiles et indolores, de 1,5 cm de diamètre, siégeant sur les régions épitrochléenne et axillaire homolatérales. Le reste de l’examen clinique était normal. Les sérologies virales hépatiques, tréponémiques et rétrovirale (VIH) étaient négatives. L’examen histologique d’un fragment biopsique confirmait le diagnostic de carcinome épidermoïde bien différencié et infiltrant. La biopsie de l’adénopathie axillaire objectivait une métastase ganglionnaire avec effraction de la capsule et extension au tissu péri-ganglionnaire. Le reste du bilan d’extension était sans anomalie. Une exérèse large de la lésion avec curage ganglionnaire étaient pratiqués, compléter par une radiothérapie. L’évolution était marquée par une récidive locale précoce (deux mois plus tard) nécessitant une reprise chirurgicale. Les suites opératoires étaient simples.

**Figure 1:**
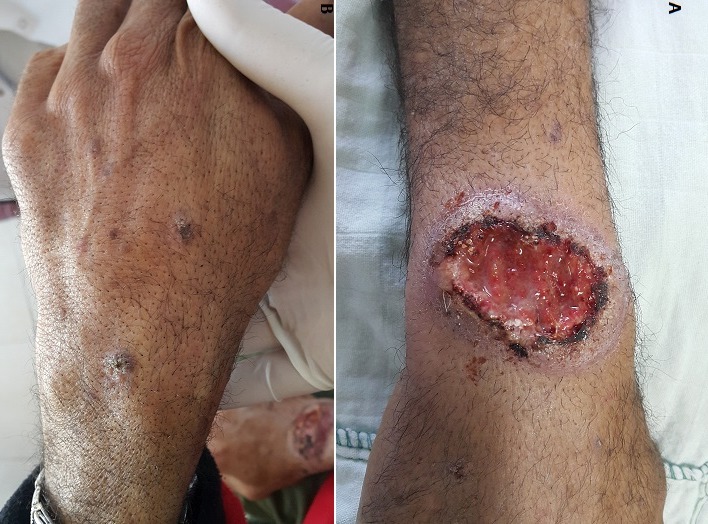
A) tumeur ulcéro-bourgeonnante sur la face dorsale du poignet droit, mesurant 5 × 6 cm; B) multiples lésions de kératose actinique diffuses sur le dos de la main gauche

